# DAKS1, a Kunitz Scaffold Peptide from the Venom Gland of *Deinagkistrodon acutus* Prevents Carotid-Artery and Middle-Cerebral-Artery Thrombosis via Targeting Factor XIa

**DOI:** 10.3390/ph14100966

**Published:** 2021-09-24

**Authors:** Zhiping Jia, Yunyang Liu, Xiaoru Ji, Yizheng Zheng, Zhengyang Li, Shuai Jiang, Hongjin Li, Yi Kong

**Affiliations:** School of Life Science and Technology, China Pharmaceutical University, 24 Tong Jia Street, Nanjing 210009, China; 3219030696@stu.cpu.edu.cn (Z.J.); 1821030696@stu.cpu.edu.cn (Y.L.); 1822030732@stu.cpu.edu.cn (X.J.); 3219030707@stu.cpu.edu.cn (Y.Z.); 3220030542@stu.cpu.edu.cn (Z.L.); 3219030711@stu.cpu.edu.cn (S.J.); 2020181453@stu.cpu.edu.cn (H.L.)

**Keywords:** Kunitz, scaffold-based peptides, *Deinagkistrodon acutus*, DAKS1, Factor XIa, thrombosis

## Abstract

Scaffold-based peptides (SBPs) are fragments of large proteins that are characterized by potent bioactivity, high thermostability, and low immunogenicity. Some SBPs have been approved by the FDA for human use. In the present study, we developed SBPs from the venom gland of *Deinagkistrodon acutus* (*D. acutus*) by combining transcriptome sequencing and Pfam annotation. To that end, 10 Kunitz peptides were discovered from the venom gland of *D. acutus*, and most of which peptides exhibited Factor XIa (FXIa) inhibitory activity. One of those, DAKS1, exhibiting strongest inhibitory activity against FXIa, was further evaluated for its anticoagulant and antithrombotic activity. DAKS1 prolonged twofold APTT at a concentration of 15 μM in vitro. DAKS1 potently inhibited thrombosis in a ferric chloride-induced carotid-artery injury model in mice at a dose of 1.3 mg/kg. Furthermore, DAKS1 prevented stroke in a transient middle cerebral-artery occlusion (tMCAO) model in mice at a dose of 2.6 mg/kg. Additionally, DAKS1 did not show significant bleeding risk at a dose of 6.5 mg/kg. Together, our results indicated that DAKS1 is a promising candidate for drug development for the treatment of thrombosis and stroke disorders.

## 1. Introduction

Scaffold-based peptides are fragments of large proteins that play an important role in biological processes, and are characterized by high thermostability and low immunogenicity [1,2,3]. Their structures are similar to those of antibodies; each scaffold includes a constant region, which provides a stable secondary structure, and one or more variable loops, which are a response for binding to given targets. Some of these SBPs were approved by the FDA for human use [4,5], and many are currently being evaluated preclinically or in clinical trials, such as Kunitz domains, adnectins, anticalins, avimers, Fynomers, knottins, affibodies, β-hairpin mimetics, and DARPins [2,6].

Kunitz domains are derived from the active motif of Kunitz-type protease inhibitors, and they mainly inhibit the function of protein-degrading enzymes or, more specifically, they are serine protease inhibitors [7,8,9]. Kunitz SBPs are relatively small with a length of about 50 to 60 amino acids and a molecular weight of 6–7 kDa [10]. Ecallantide, a Kunitz scaffold-based inhibitor of kallikrein, was approved by the FDA in 2012 for the treatment of hereditary angioedema [11,12,13]. Depelstat, a Kunitz scaffold-based peptide, is a potent and selective inhibitor of human neutrophil elastase that mediates inflammation and contributes to lung damage in cystic fibrosis [14,15]. Depelstat can reduce neutrophil transepithelial migration and inflammation ex vivo [16]. Therefore, Kunitz SBPs are promising compounds for the treatment of serine protease-related diseases.

FXIa is an important serine protease, and plays a significant role in the intrinsic pathway [17]. Recent clinical studies showed that patients with FXI deficiency had reduced incidence of deep-vein thrombosis and ischemic stroke [18,19]. FXI^−/−^ mice were protected from FeCl_3_-induced carotid-artery thrombus formation [20,21,22]. This evidence supports that FXI is a good target for antithrombotic drug discovery. Many Kunitz scaffold peptide inhibitors targeting FXIa were discovered in recent years, such as PN2KPI from the human platelet [23,24], Fasxiator from *Bungarus fasciatus* [25], and Ir-CPI from *Ixodes ricinus* [26,27].

In the current study, we developed Kunitz SBPs from the venom gland of *D. acutus* by combining transcriptome sequencing and Pfam annotation. To that end, 10 Kunitz SBPs were discovered from the venom gland of *D. acutus*. These peptides were cloned and expressed, and their inhibitory activity against FXIa was tested. DAKS1, which exhibited the strongest inhibitory activity towards FXIa, was further assessed for its anticoagulant activity in vivo.

## 2. Results

### 2.1. Ten Kunitz SBPs Discovered from Venom Gland of D. acutus

**Figure 1:**
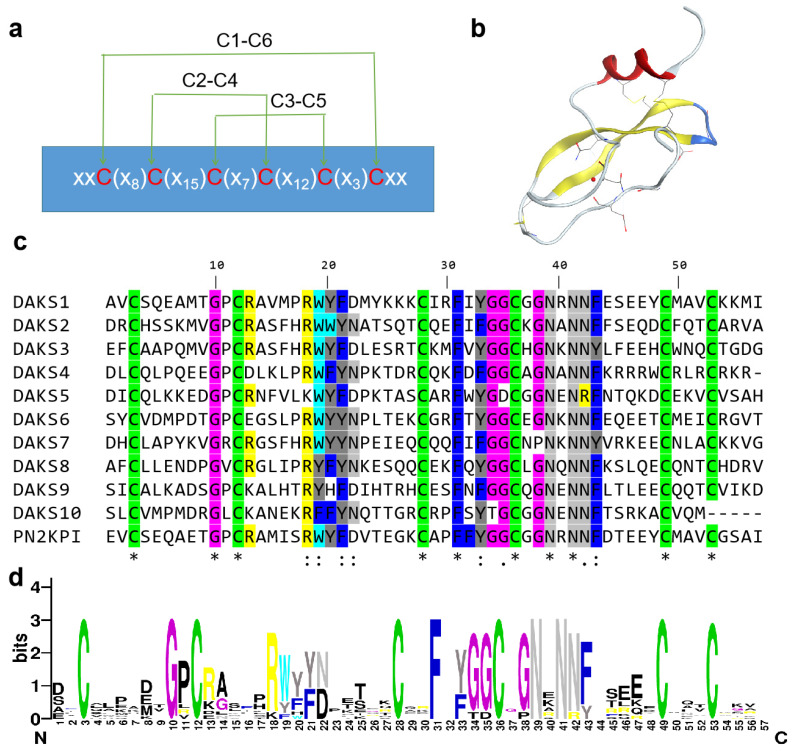
Structure and sequence characteristics of Kunitz peptides DAKS1-10. (**a**) Schematic representation of disulfide bonding pattern of Kunitz domains. Conserved cysteine residues are marked with red. “x” represents random amino acid residues. (**b**) Crystal structure of PN2KPI (PDB: 1ZJD). Antiparallel β-sheets are marked in yellow, alpha helical is marked in red., and β-turn is marked in blue. (**c**) Alignment of DAKS1-10 and PN2KPI. “*” indicates fully conserved amino acid residue, “:” indicates amino acid residue with similar properties, and “.” indicates amino acid residue with weak similar properties. (**d**) Sequence logo of amino acid sequences of DAKS1-10. Logo represents each column of the alignment in a stack of letters, with the height of each letter proportional to the observed frequency of the corresponding amino acid residue, and the overall height of each stack proportional to the sequence conservation.

The transcriptome of the venom gland of *D. acutus* was analyzed using Illumina Hiseq 4000 sequencing, and 45,321 unigenes with length distribution N50 = 1462 were discovered. Unigenes were annotated by using the GO, COG, KEGG, Nr, Nt, Swissprot, and Interpro databases, separately, and 11 full-length Kunitz proteins (Table S1) were selected from the annotated transcriptome library on the basis of a “Kunitz” keyword search. The 12 Kunitz SBPs sequences in these proteins were recognized by the Pfam database. When the E-value was below 10^−15^, 2 Kunitz peptides sequences were screened out, and 10 classical Kunitz peptides remained (Figure S1a,b); those Kunitz peptides were named DAKS1-10 (Table 1 and Figure 1c). The conserved amino acids in the DAKS1-10 peptides are shown in Figure 1d. Classical Kunitz domains consist of 50–60 amino acids and adopt a conserved structural fold with two antiparallel β-sheets and one or two alpha helical regions that are stabilized with three highly conserved disulfide bridges with the bonding pattern of C1–C6, C2–C4, and C3–C5 [7,28,29] (Figure 1a,b).

### 2.2. FXIa Inhibitory Activity of 10 Kunitz Peptides Tested by Substrate Chromogenic Method

**Figure 2:**
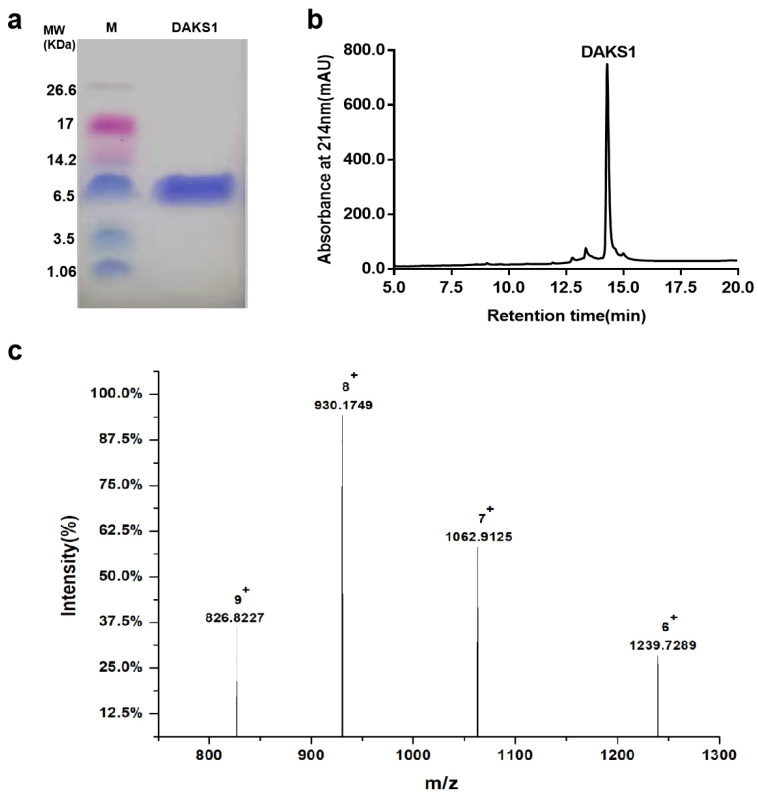
Characterization of DAKS1. (**a**) Tricine–SDS-PAGE analysis of DAKS1. (**b**) HPLC analysis of DAKS1. (**c**) LC–Q-TOF–MS analysis of DAKS1.

To test whether DAKS1-10 has inhibitory activity towards FXIa, genes of DAKS1-10 were cloned into the pPIC9k, and were expressed using *Pichia pastoris* GS115. After purification by using ammonium sulfate precipitation and affinity chromatography on a UniNTA-80Ni column, the inhibitory activity of DAKS1-10 towards FXIa was tested with the substrate chromogenic method. Results showed that most of the Kunitz domains can inhibit FXIa; one of those, DAKS1, had the strongest inhibitory activity (Table 2). The yield of DAKS1 was 10 mg peptides per liter of fermentation broth. It showed one band in Tricine–SDS-PAGE analysis (Figure 2a), and showed a purity of 90% in HPLC analysis (Figure 2b). LC–Q-TOF–MS indicated that the MW of DAKS1 was 7434.3658 ± 0.0174 Da (Figure 2c), which is identical to the theoretical MW of 7434.35 Da.

### 2.3. DAKS1 Is a Potent FXIa Inhibitor

**Figure 3:**
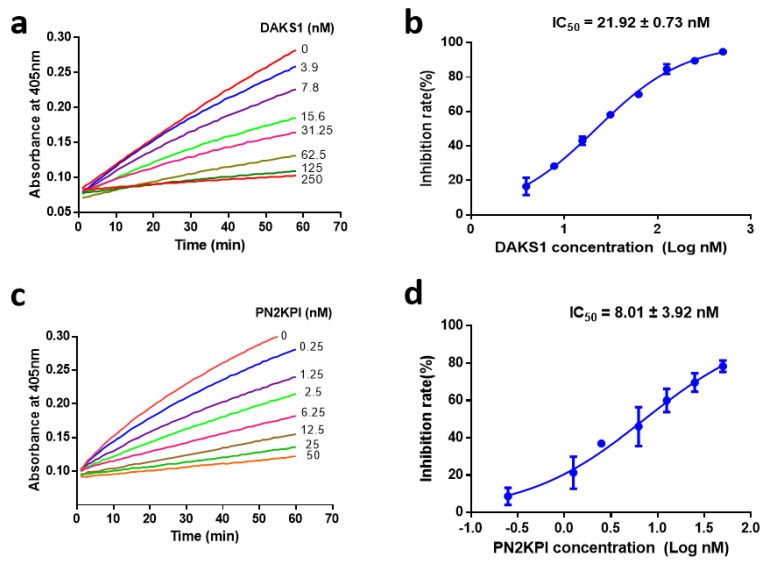
DAKS1 is a potent inhibitor of FXIa. (**a**) Inhibitory activity of DAKS1 towards FXIa. Reactions began by the addition of CS-21 (66) (250 μM) to a mixture containing DAKS1 (0–250 nM) incubated for 60 min with FXIa (0.5 nM). Substrate hydrolyzed and monitored at 405 nm for 60 min. (**b**) Determination of IC_50_ of DAKS1. Plot of inhibition rate (%) vs. log DAKS1 concentrations fitted by nonlinear regression, and value of IC_50_ was obtained by fit log(inhibitor) vs. normalized response-variable slope. (**c**) Inhibitory activity of PN2KPI towards FXIa. Reactions began by the addition of CS-21 (66) (250 μM) to a mixture containing PN2KPI (0–50 nM) incubated for 60 min with FXIa (0.5 nM). Substrate was hydrolyzed and monitored at 405 nm for 60 min. (**d**) Determination of IC_50_ of PN2KPI. Plot of inhibition rate (%) vs. log PN2KPI concentrations fitted by nonlinear regression, and value of IC_50_ was obtained by fit log(inhibitor) vs. normalized response-variable slope. Data presented as mean ± standard deviation (SD) of three independent experiments.

DAKS1 inhibited the amidolytic activity of FXIa towards its substrate S-21 (66) in a dose-dependent manner with an IC_50_ value of 21.92 ± 0.73 nM (Figure 3a,b). We tested the inhibitory activity of DAKS1 to other serine proteases, such as thrombin, FXIIa, FXa, kallikrein, and trypsin, and results showed that DAKS1 did not exhibit inhibitory activity to thrombin and FXIIa. DAKS1 had little inhibitory function towards FXa and kallikrein when a 1:125 molar ratio of FXIa and DAKS1 resulted in efficient FXIa inhibition of 70%, whereas a molar ratio of 1:6000 of FXa and DAKS1, and 1:3000 of kallikrein and DAKS1 resulted in FXa and kallikrein inhibition of 27.45% and 62.28%, respectively (Table 3). However, DAKS1 had strong inhibitory function to trypsin with an IC_50_ value of 34.26 nM (Figure S2a,b). Classical Kunitz peptide PN2KPI secreted from the human platelet [23,24] was cloned and expressed in our laboratory, and its inhibitory activity towards various serine proteases was also determined. PN2KPI had potent inhibitory activity towards FXI and trypsin with IC_50_ values of 8.01 ± 3.92 nM (Figure 3c,d) and 25.01 ± 4.94 nM (Figure S2c,d), respectively, and showed no or weak inhibitory activity towards other serine proteases (Table 3).

Inhibition of DAKS1 against FXIa, FXa, thrombin, kallikrein, trypsin, and FXIIa assessed in chromogenic substrate assay. Inhibitor was incubated with the respective protease at the indicated molar ratio in the presence of a specific protease substrate. Results obtained from 3 independent experiments. PN2KPI served as control inhibitor.

### 2.4. DAKS1 Prolonged APTT in Mouse Plasma

**Figure 4:**
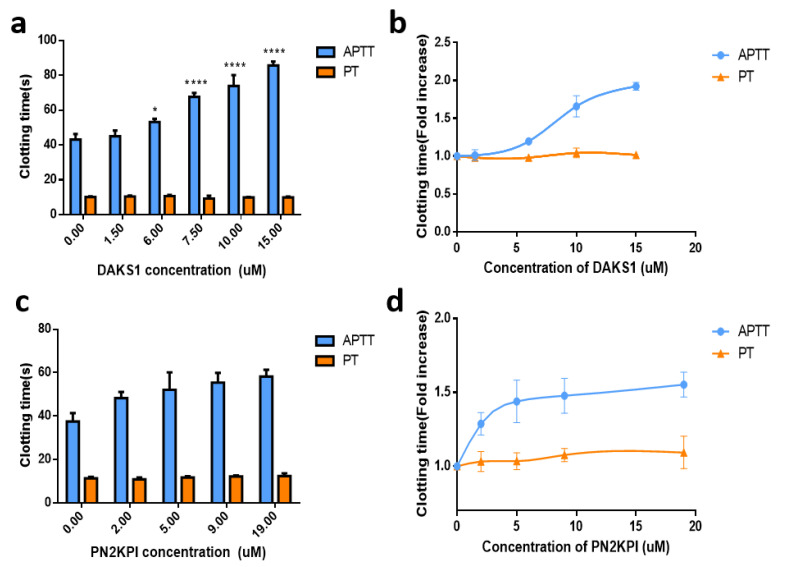
DAKS1 prolonged plasma coagulation time. Effects on APTT and PT of DAKS1 and PN2KPI evaluated using platelet-poor plasma (PPP). (**a**) DAKS1 significantly prolonged APTT in a dose-dependent manner at a concentration ranging from 1.5 to 15 μM. (**b**) DAKS1 could prolong APTT by about 2 times, but not prolong PT at the same concentration. (**c**) PN2KPI significantly prolonged APTT in a dose-dependent manner at a concentration ranging from 2 to 19 μM. (**d**) PN2KPI could prolong APTT by about 1.5 times, but not prolong PT at 20 μM. Data presented as mean ± SD of three independent experiments. **** *p* < 0.0001 versus vehicle, * *p* < 0.05 versus vehicle by one-way ANOVA and Dunnett’s multiple-comparisons test, and vehicle means a lack of addition of DAKS1 or PN2KPI.

FXIa belongs to intrinsic coagulation factors, and its inhibition leads to the prolongation of APTT, and not PT [30,31]. DAKS1 significantly prolonged APTT in a dose-dependent manner at a concentration ranging from 1.5 μM to 15 μM. As shown in Figure 4a,b, APTT and PT in the saline-treated group were 43.13 ± 3.13 s and 10.15 ± 0.35 s (mean ± SD, *n* = 3), respectively. In the DAKS1-treated groups, APTT significantly increased to 85.63 ± 2.32, 73.87 ± 6.25, and 67.60 ± 2.26 s at doses of 15, 10, and 7.5 μM (*p* < 0.0001 versus saline), respectively. For PT, at a high tested dose of 15 μM, DAKS1 did not significantly prolong its time when compared to the saline group (*p* > 0.05). PN2KPI significantly prolonged APTT in a dose-dependent manner at a concentration ranging from 2 to 19 μM. At doses of 5, 9, and 19 μM, the APTT were 52.10 ± 8.06, 55.90 ± 5.32, and 59.40 ± 2.62 s (*p* < 0.0001 versus saline), respectively (Figure 4c,d).

### 2.5. DAKS1 Inhibited Ferric Chloride-Induced Carotid-Artery Thrombosis in Mice

**Figure 5:**
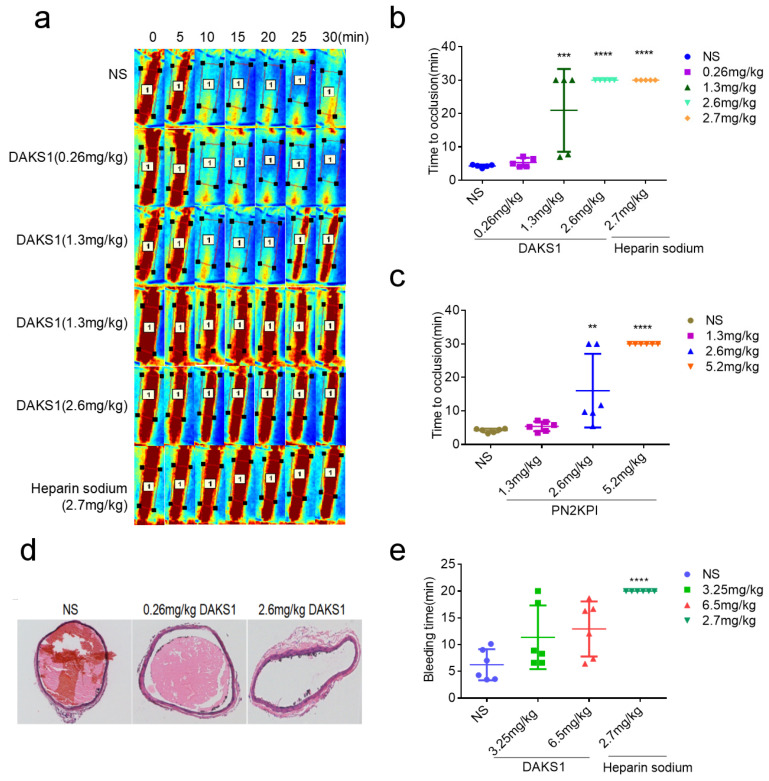
DAKS1 inhibited thrombosis in a FeCl_3_-induced carotid-artery injury model. Ten min after FeCl_3_-induced injury, DAKS1 (0.26, 1.3, and 2.6 mg/kg) and heparin sodium (2.7 mg/kg) were injected by tail vein. (**a**) After treatment with 6% FeCl_3_, blood flow at region of interest (ROI) was monitored at 0, 5, 10, 15, 20, 25, and 30 min. (**b**) Effects of DAKS1 (0.26, 1.3, and 2.6 mg/kg), and heparin sodium (2.7 mg/kg) on the inhibition of thrombosis. (**c**) Effects of PN2KPI (1.3, 2.6, and 5.2 mg/kg) on inhibition of thrombosis. (**d**) H&E staining micrographs of carotid-artery samples from a control mouse and the mouse administered with DAKS1 (0.26 and 2.6 mg/kg). (**e**) Bleeding time of DAKS1 in mice. Fifteen minutes after the administration of DAKS1 (3.25, 6.5 mg/kg), heparin sodium (2.7 mg/kg) and the NS, a 2 mm long tail tip was cut from the mice, and the remaining tail was immersed immediately into saline at 37 °C. Accumulated bleeding time (including periods of rebleeding) was recorded over a 20 min period. NS: normal saline. Data presented as mean ± SD (*n* = 6). ** *p* < 0.05, *** *p* < 0.001, **** *p* < 0.0001 versus NS, analyzed by one-way ANOVA, followed by Dunnett’s multiple comparison test.

The effect of DAKS1 on thrombosis was evaluated by a ferric chloride-induced carotid-artery injury model in C57BL/6J male mice. Heparin sodium, which is clinical antithrombotic medicine, was used as a positive control. As shown in Figure 5a,b, times to occlusion were not significantly different between vehicle group (4.29 ± 0.43 min, mean ± SD, *n* = 5) and 0.26 mg/kg DAKS1-treated group (5.36 ± 1.37 min, mean ± SD, *n* = 5). DAKS1, at a dose of 1.3 mg/kg, significantly prolonged times to occlusion (20.95 ± 12.40 min, mean ± SD, *n* = 5). Occlusion did not happen within 30 min in three mice. While occlusion appeared at about 6 minutes in the two other mice, and more than 1 minute later, the thrombosis was flushed away. At a dose of 2.6 mg/kg, no DAKS1-treated mice formed occlusive thrombi within 30 min (30.00 ± 0.00 min, mean ± SD, *n* = 5), which was similar to that of heparin sodium. At a dose of 2.6 mg/kg of PN2KPI, occlusion did not happen within 30 min in two mice, and the occlusion appeared in three mice. At a high dose of 5.2 mg/kg, PN2KPI produced a similar effect as that of DAKS1 at 2.6 mg/kg (Figure 5c and Figure S3a,b). The vessel sections of injured carotid artery stained by hematoxylin and eosin (H&E) showed that the thrombi in the lumen of the vessel of DAKS1-treated mice were smaller than those that appeared in vehicle group (Figure 5d).

### 2.6. DAKS1 Inhibited Stroke in Mice

**Figure 6:**
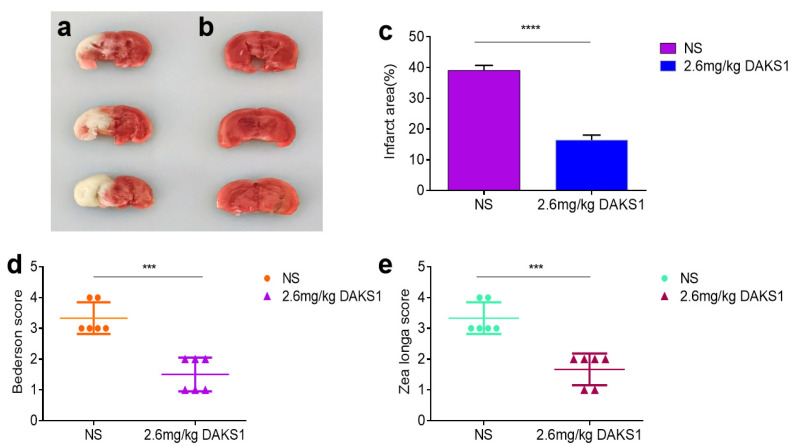
DAKS1 protected from ischemic stroke in mice. Cerebral ischemia and neurological damage evaluated in a tMCAO model. (**a**) Representative coronal brain sections 24 h after tMCAO from mice treated with saline. Pale area represents ischemic infarctions. Mice treated with DAKS1 (**b**). (**c**) Brain infarct area measured by image J (*n* = 6). (**d**,**e**) Bederson and Zea Longa scores in respective groups after 24 h of tMCAO. Data represent mean ± SD (*n* = 6), *** *p* < 0.001, **** *p* < 0.0001 by unpaired *t*-Test.

A tMCAO model in mice was used to evaluate the protective potency of DAKS1. As shown in Figure 6a,b, DAKS1 at 2.6 mg/kg significantly reduced the cerebral infarct area compared with those from a control group. The cerebral infarct area decreased from ~39.07% to ~16.45% (Figure 6c). Zea Longa and Bederson scores were used to assess neurological function, and results showed that DAKS1 could reduce ischemic stroke (Figure 6d,e).

### 2.7. DAKS1 Did Not Show Bleeding Risk in Mice

The bleeding risk of DAKS1 was measured by mouse-tail cutting assay. Doses of DAKS1 were designed as 3.25 and 6.5 mg/kg, which represent 2.5 and 5 times the efficient dose (1.3 mg/kg) used for ferric chloride-induced carotid-artery thrombosis study, and heparin sodium (2.7 mg/kg) was used as a positive control. As shown in Figure 5e, DAKS1 did not significantly prolong bleeding time, even at a high dose of 6.5 mg/kg (12.93 ± 5.14 min, mean ± SD, *n* = 6), compared with the vehicle group (6.26 ± 2.90 min, mean ± SD, *n* = 6), but heparin-treated mice were unable to stop tail bleeding within 20 min (>20.00 ± 0.00 min, mean ± SD, *n* = 6).

## 3. Discussion

We discovered 10 Kunitz scaffold peptides from the venom gland of *D. acutus*, and most of those peptides exhibited FXIa inhibitory activity, determined by the chromogenic substrate method. DAKS1, exhibiting strongest inhibitory activity against FXIa, was further evaluated for its anticoagulant activity. Results showed that DAKS1 prolonged APTT, potently inhibited thrombosis formation in a ferric chloride-induced carotid thrombus formation model, and strongly prevented stroke in a tMCAO model. Interestingly, this Kunitz peptide did not significantly prolong bleeding time, even at five times the effective antithrombotic dosage in a mouse-tail cutting assay, indicating that DAKS1 is a promising candidate for drug development for the treatment of thrombosis and stroke disorders.

Protein scaffolds are a wide research focus because they can be used as antibody replacement therapy [32,33,34,35]. Scaffold-based specific peptides are usually developed by using display technologies, such as phage, yeast, or ribosome/mRNA displays [36]. With these technologies, a peptide library is built by the artificial random assembly of amino acids, but scaffolds used in drug studies are originally born from natural proteins that play an important role in biological processes [37,38,39]. Biological evolution should be a more rational method for producing bioactivity protein scaffolds. Snake venoms are a weapon library formed through millions of years of adaptive evolution for their prey and defense in the natural environment [40,41]. *D. acutus* is a poisonous snake, and its envenomation incurs hemorrhage and thrombus formation [42]. The venom contains an activator or inhibitor of the coagulation system, especially Kunitz/BPTI-type serine protease inhibitors [43,44]. Kunitz domains usually consist of 50–60 amino acids, and some are serine protease inhibitors. Ecallantide [45,46], a Kunitz scaffold-based inhibitor of kallikrein, was approved by the FDA in 2012 for the treatment of hereditary angioedema. In the present study, we combined transcriptome sequencing and Pfam annotation to reveal Kunitz scaffold peptides in *D. acutus* venom, and 10 Kunitz peptides were found. Those 10 Kunitz peptides were then cloned and expressed in *P. pastoris*, and inhibitory activity towards FXIa, a serine protease, was tested. Results showed that most of them could inhibit FXIa. DAKS1, which exhibited the strongest inhibitory activity against FXIa. The systemic bioactivity study of DAKS1 was further performed, including other coagulant factors inhibition in vitro, and antithrombosis and preventing stroke in vivo.

DAKS1 displayed potent inhibition activity to FXIa with an IC_50_ value of 21.92 ± 0.37 nM, had strong inhibitory action to trypsin, and had no or weak inhibitory activity on FXa, FXIIa and thrombin. Fasxiator [25] is a 7 kDa Kunitz scaffold-based peptide from the venom of the *Bungarus fasciatus* snake with weaker inhibitory activity (IC_50_ = 1.5 μM) than that of DAKS1, while Desmolaris [30], a Kunitz domain protein from the salivary gland of a vampire bat, has poor selectivity towards coagulation factors (K_i_ for FXIa = 12.35 nmol L^–1^, K_i_ for FXa = 15.06 nmol L^–1^). Ir-CPI, a Kunitz peptide from *Ixodes ricinus*, binds both to FXIa, and to FXIIa and plasma kallikrein [26]. PN2KPI [23,47], a Kunitz peptide secreted from platelet [24], is a potent inhibitor of FXIa. DAKS1 showed 68% identity with PN2KPI, aligned by using BLAST. PN2KPI was cloned, expressed, and purified as a control in the present study (Figure 1c), and its IC_50_ value to FXIa was 8.01 ± 3.92 nM, as tested in our laboratory. The anticoagulant activity of DAKS1 and PN2KPI was further evaluated by using APTT and PT assays. Compared with the negative control, DAKS1 prolonged twofold APTT at a concentration of 15 μM. However, PN2KPI only prolonged 1.5-fold APTT, even if its concentration was increased to 19 μM, indicating that, though the inhibitory activity of DAKS1 towards FXIa was weaker than that of PN2KPI in the amidolytic activity test of the enzyme, the ability of DAKS1 for prolonging APTT was stronger than that of PN2KPI.

Ferric chloride-induced carotid thrombus formation model in mice was used to evaluate the antithrombotic properties of DAKS1 and PN2KPI. Results showed that DAKS1 could inhibit thrombus formation in a dose-dependent manner. At a concentration of 2.6 mg/kg, DAKS1 thoroughly inhibited thrombus formation in carotid-artery vessels, which was similar to heparin sodium, antithrombotic medicine widely used in clinics. PN2KPI, at a dose of 2.6 mg/kg, partially inhibited thrombi formation; at a higher dose of 5.2 mg/kg, it could totally inhibit thrombus formation, indicating that DAKS1 exerted stronger antithrombotic activity than that of PN2KPI in vivo, which is consistent with results in the APTT assay. The effect of DAKS1 on thrombosis was further investigated by using a transient cerebral-ischemia model in mice. DAKS1 reduced the cerebral infarct area. The Bederson score is a standard method to measure neurological deficits following stroke according to resistance to lateral push, forelimb flexion, and circling behavior [48]. The Zea Longa scoring method is similar to the Bederson method. Scores were measured as follows: 0, no symptom of neurological impairment; 1, the contralateral forelimb is unable to contract when the tail is lifted; 2, inward rotation when walking; 3, tilted inwards when walking; 4, fails to spontaneously walk and loss of consciousness [49]. Neurologically impaired animals have a higher score than that of non-neurologically impaired animals. Results of Bederson and Zea-longa scores indicated that DAKS1 protected from neurological impairment. A number of antithrombotic drugs are associated with bleeding complications [50,51]. DAKS1 did not show significant bleeding risk at 5 times the dose of the efficient dose used for in vivo antithrombotic studies. These results indicated that DAKS1 may be an excellent candidate for the development of clinical antithrombotic and anti-ischemic stroke drugs.

## 4. Materials and Methods

### 4.1. Materials and Reagents

Factor Xa, thrombin, Factor XIIa, Pefachrome FXIIa/TH 5253 (H-D-CHA-Gly-Arg-pNA·2AcOH), CS-11 (22) (Bz-Ile-Glu (γOCH3)-Gly-Arg-pNa and Bz-Ile-Glu (γOH)Gly-Arg-pNa), CS-31 (02) (D-Pro-Phe-Arg-pNa, 2HCl), CS-21 (66) (p-Glu-Pro-Arg-pNa·HCl) and CS-01 (38) (H-D-Phe-Pip-Arg-pNa, 2HCl) were from Hyphen-Biomed (Neuville Sur Oise, France); HFXIa was from Enzyme Research Laboratories, (South Bend, IN, USA); kallikrein was from AssayPro LLC, (St. Charles, MO, USA). APTT and PT reagents were from Taizhou Zhongqinshidi Biotechnology Co., Ltd., (Beijing, China); FeCl_3_ was from Sinopharm Chemical Reagent Co., Ltd. (Shanghai, China); TTC was from Sigma (T8877-5G, St. Louis, MO, USA), All other chemicals used in this study were of analytical grade.

### 4.2. Animals Samples

C57BL/6J male mice were obtained from the Comparative Medicine Centre of Yangzhou University (Yangzhou, Jiangsu province, China). All animals were housed under controlled temperature (21–25 °C) and light (12 h light, 12 h dark), with ad libitum access to food and water for one week before experiments. All experiments were performed according to the guidelines and the regulations of the Ethical Committee of China Pharmaceutical University (CPU2019-S020, 19 December 2019).

### 4.3. Preparation of Venom Gland of D. acutus

The adult *D. acutus* was captured in the Yujiang Caihong snake farms (Yujiang county, Yingtan city, Jiangxi province, China). The snake was allowed to rest for three days to maximize transcription. Venom glands were promptly removed, washed in DNase/RNase-Free, and then placed into a tube with 10 mL TRI Reagent (Sigma, St. Louis, MO, USA). The tube was sent to the Beijing Genomic Institute, BGI (Shenzhen, China) for transcriptome sequencing.

### 4.4. Preparation of RNA Library and Sequencing

The library for the whole RNA sequencing was prepared through a standard protocol established by the Beijing Genomic Institute, BGI (Shenzhen, China): enrich mRNA with magnetic beads with Oligo (dT), synthesize the first-strand cDNA using the interrupted mRNA as a template, and then synthesize the second-strand cDNA. 3’ DNA adaptor was ligated to the digested DNA fragments, and products were amplified using PCR. Lastly, RNA deep sequencing was performed on the Illumina Hiseq automatic sequencing platform.

### 4.5. Bioinformatic Processing

The RNA sequencing, assembly, and assessment of *D. acutus* were as previously described [29]. The raw reads were processed to obtain clean reads by removing adapter and low-quality sequences. Next, de novo transcriptome assembly was carried out using the Trinity program (v2.0.6). TIGR Gene Indices Clustering Tools (TGICL, v2.0.6) software was used to obtain the longest and most complete consensus transcripts by clustering the assembled datasets. Blast (v2.2.23) was used to annotate unigenes with Nt, Nr, COG, KEGG and SwissProt, Blast2GO (v2.5.0), Nr annotation results were used to GO annotate, and InterProScan5 (v5.11–51.0) was used to InterPro annotate. According to the results of functional annotation, the best-comparison fragments of unigenes were selected as the CDS of the unigene according to the database priority order of Nr, SwissProt, KEGG, and COG. Unigenes on the failed annotations were modeled by using the CDS predicted in the previous step, and predicted using ESTScan (v3.0.2). On the basis of the functional annotation, we searched proteins with a “Kunitz” keyword search in the annotated transcriptome library.

### 4.6. Pfam Platform Recognizing

Pfam is a sequence-based classifications database, that provides an alternative classification based on evolutionarily conserved repeat families [52]. Kunitz proteins were searched in the “sequence” box of Pfam database to recognize the specific Kunitz peptide sequences using hidden Markov models (HMMs). In the Pfam database, expectation (E-) values are calculated. An E-value is the number of hits that are expected to have a score equal to or better than this value by chance alone, and a good E-value is much less than 1 [53]. So, in this study, in order to obtain sequences that were highly consistent with the Kunitz domain feature, the E-value was limited to less than or equal to 10^−15^.

### 4.7. Expression and Purification of Kunitz Peptides

The genes of all Kunitz peptides were synthesized and seamlessly cloned into the pPIC9k. Plasmids were linearized with the Sac I restriction enzyme (TaKaRa). Linearized plasmids were then transformed into competent *P. pastoris* GS115 cells using an electroporator (Bio-Rad, Hercules, CA, USA). Then, they were spread onto MD plates, and G418-resistant colonies were verified by PCR. Verified clones were cultured in BMGY (1% yeast extract, 1.34% YNB, 2% peptone, 1% glycerol, 100 mM sodium phosphate buffer pH 6, 4 × 10^−5^% biotin) at 28.5 °C until suitable cell density was achieved. These cells were transferred to BMMY (1% yeast extract, 1.34% YNB, 2% peptone, 1% methanol, 100 mM sodium phosphate buffer pH 6, 4 × 10^−5^% biotin), and cultured at 28.5 °C for 72 h, and 1% methanol was supplemented every 24 h to induce protein express. Yeast cultures containing Kunitz proteins were centrifuged to remove yeast cells. The supernatant was precipitated using 80% saturation concentration of ammonium sulfate and then centrifuged at 13,656× *g* at 4 °C for 30 min. Precipitates were resuspended in 20 mM NaH_2_PO_4_–Na_2_HPO_4_ buffer (pH 7.0). Samples were dialyzed in a dialysis bag with a molecular cut-off 3.5 KDa (Millpore) in a 20 mM NaH_2_PO4–Na_2_HPO4 (pH 7.0) at 4 °C, and then loaded into a UniNTA-80Ni column (Nanomicro, Suzhou, China). The column was washed with 100 mL of NaH_2_PO_4_–Na_2_HPO_4_ buffer (pH 7.0), followed by 50 mL each of 20, 40, 200, and 600 mM imidazole diluted in a NaH_2_PO_4_–Na_2_HPO_4_ buffer (pH 7.0). Fractions eluted by 200 mM imidazole solution were collected and dialyzed against 20 mM NaH_2_PO_4_–Na_2_HPO_4_ buffer (pH 7.0), and then concentrated with 3 kDa ultrafiltration tubes (Millipore, Bedford, MA, USA). Sample concentration was estimated by BCA protein assay kit (Beyotime), and peptide yield was calculated by the concentration of the peptide multiplied by the volume, and then divided by the volume of the fermentation broth. Peptide purity was analyzed by Tricine–SDS-PAGE.

### 4.8. HPLC

DAKS1 purity was analyzed by HPLC on an Agilent ZORBZAX 300 SB-C18 column (4.6 mm × 250 mm) under linear gradient elution conditions by using acetonitrile as the organic modifier, and trifluoroacetic acid (TFA) as the volatile buffer. Eluent A consisted of 0.1% TFA in distilled water (*v/v*), and eluent B consisted of 0.1% TFA in 100% acetonitrile (*v/v*). Gradient elution was carried out according to the following process: 0–20 min, B 5–50%; 20–22 min, B 50–90%; and 22–25 min, B 90%. Flow rate was 1 mL/min. UV absorbance was monitored at 214 nm.

### 4.9. LC–Q–TOF–MS Analysis

LC–Q–TOF–MS analysis was carried out using an Agilent Technologies (Santa Clara, CA, USA) 1290 Infinity Series liquid chromatograph coupled with an Agilent Technologies 6500 iFunnel Q–TOF LC/MS device equipped with an electrospray ionization Agilent Technologies Jet Stream ion source. Chromatographic separation was achieved on an Agilent ZORBZAX 300 SB-C18 column (4.6 mm × 250 mm) (Agilent, Santa Clara, CA, USA). Injection volume was 20 µL. The mobile phase consisted of 0.1% formic acid in Milli-Q water (solvent A) and 0.1% formic acid in 100% acetonitrile (solvent B) at a flow rate of 0.5 mL/min. The mobile-phase gradient (10–60% B) was applied. Source nitrogen gas temperature was 325 °C, sheath gas flow was 12 L/min, and nebulizer pressure was 40 psig. Voltages were set at 4000 (capillary) and 175 V (fragmentor). Positive ions were acquired in the range of 100–3200 *m*/*z* for MS scans. Internal mass correction was enabled by using two reference masses at 121.0509 and 922.0098 *m*/*z*. Data acquisition and instrument control were performed using Agilent MassHunter Workstation software (B.06.01 SPI).

### 4.10. FXIa Inhibitory Activity Testing

The inhibitory activities of purified Kunitz peptides to FXIa were tested as previously [41]. In brief, FXIa (100 μL) was diluted with a buffer, TBS-BSA (10 mM Tris, 0.15 M NaCl, 0.3% BSA, pH 7.4) was preincubated with 50 μL different concentrations of proteins for 60 min at 37 °C, followed by the addition of 50 μL of the chromogenic substrate. The cleavage of substrate was continuously monitored at 405 nm for 60 min using a microplate reader (FEL-1). The slopes of the absorbance–time curves (V_i_) were used to calculated the inhibitory activity of Kunitz peptides. BMMY medium was used as a control (slope of curve V_0_). The inhibitory effect was calculated according to Equation (1):

Inhibitory rate (%) = (V_0_ − V_i_)/V_0_ × 100 (1)

where V_0_ represents the slope of the control, and V_i_ represents the slope of the Kunitz peptides. IC_50_ was defined as the inhibitor concentration required to inhibit the activity of FXIa by 50%.

### 4.11. Protease Selectivity Testing

DAKS1 and serine protease (FXIa (0.5 nM), FXIIa (1 nM), kallikrein (0.5 nM), FXa (2.5 nM), and thrombin (1.42 nm)) were mixed with different molar ratios in TBS-BSA, and then incubated for 60 min at 37℃. The chromogenic substrate (CS-21 (66) (0.25 mM), TH 5253 (0.25 mM), CS-31 (02) (0.25 mM), CS-11 (22) (0.44 mM), CS-01 (38) (100 µM)) was added into the corresponding protease well, and absorbance at 405 nm was then continuously tested for 1 h using an FEL-1.

### 4.12. APTT and PT

Blood-clotting time was measured as previously described [41]. Briefly, 40 μL of PPP drawn from C57BL/6J male mice was added into test cuvettes and incubated with 10 μL DAKS1 or saline for 3 min at 37 °C. For the APTT assay, 50 μL of the APTT reagent was added and incubated for 3 min, and 50 μL prewarmed CaCl_2_ was then added to start reactions. For the PT assay, 100 μL prewarmed PT reagent was added to start the reactions. Time to clot formation was recorded in triplicate.

### 4.13. Antithrombotic Activity Assay

The antithrombotic activity of DAKS1 was evaluated by a FeCl_3_-induced carotid-artery injury model as previously described [54]. Briefly, C57BL/6J male mice were anesthetized with chloral hydrate (5%, 10 mL kg^−1^) by i.p. injection and then fixed in a supine position on a heating pad (37 °C) to maintain body temperature. An incision was made directly into the skin of the common carotid-artery region. The fascia was then bluntly dissected, and a segment of the common carotid artery was exposed. Different concentrations of DAKS1 (0.26, 1.3, or 2.6 mg/kg), heparin sodium (2.7 mg/kg), or vehicle (saline) were injected through a tail vein. The mice were then transferred to moorFLPI-2 (model no: moorFLPI-2, Moor Instruments Limited, Millwey, Axminster, Devon, EX13 5HU, UK) and appropriately positioned under the laser. Ten minutes later, thrombus formation was induced by applying a piece of filter paper (1 mm × 2 mm) saturated with 6% FeCl_3_ solution on the adventitial surface of the artery. Three minutes later, the filter paper was removed. The carotid artery was washed with saline three times. Blood flow was continuously monitored from the onset of injury until stable occlusion occurred or for 30 min if occlusion did not occur. Data were analyzed by moorFLPIReviewV50 software.

### 4.14. Transient Occlusion Model of Middle Cerebral Artery

The tMCAO model was used to induce focal cerebral ischemia as previously described [55]. Mice (C57BL/6J, male, 22–25 g) were anesthetized and fixed in a supine position on a heating pad to maintain at 37 °C during the whole period of surgery. An incision was made directly into the skin of the common carotid artery region, the proximal common carotid artery and external carotid artery (ECA) were ligated, and a polylysine-coated nylon monofilament (A4-162020, Beijing Xinong, China) was inserted from the common carotid artery to the left internal carotid artery to occlude the origin of the left middle cerebral artery. One hour later, DAKS1 (2.6 mg/kg) was injected by tail vein. Ten minutes later, the occluding filament was removed to allow for reperfusion. After 24 hours, mice were sacrificed, and the brain was quickly removed, frozen for 20 min, and was cut into 2 mm thick coronal sections. Brain sections were then stained with 2% TTC (T8877-5G, Sigma, St. Louis, MO, USA). Stained slices were photographed and then analyzed by ImageJ software to quantify the infarcted area [56]. Bederson [48,57] and Zea Longa [49,58] scores were used to estimate neurological function.

### 4.15. Tail Bleeding-Time Assay

Bleeding time in mice was evaluated by a tail-cutting model as previously described [59]. Mice were randomly divided into four groups, namely, a vehicle (saline) group, a positive group (2.7 mg/kg of heparin sodium), and two sample groups (DAKS1 3.25 and 6.5 mg/kg). After steadily placing the anesthetized mice, the tail was washed with 75% ethanol. Samples and vehicle were injected by tail vein. Ten minutes later, the distal 2 mm segment of the tail was transected with a scalpel, and the tail was immediately immersed in 12 mL 0.9% saline warmed to 37 °C. The time was recorded until the cessation of the stream of blood. If the bleeding did not stop after 20 min, bleeding time was recorded as 20 min.

### 4.16. Statistical Analysis

Data were analyzed by using GraphPad Prism 6 (GraphPad Software, Inc., La Jolla, CA, USA). Results are expressed as mean ± SD values. The statistical significance of a two-sample comparison was evaluated by using an unpaired *t*-test, while multiple-sample comparisons were evaluated by using one-way ANOVA analysis, tested by Dunnett’s multiple comparisons. *p* < 0.05 was considered to be statistically significant.

## 5. Conclusions

In conclusion, by combining transcriptome sequencing and Pfam annotation, 10 Kunitz scaffold peptides were revealed from the venom of *D. acutus*. DAKS1, one of those peptides, showed potent inhibitory activity against FXIa, and inhibited thrombosis, preventing cerebral infarction in vivo without bleeding risk.

## Figures and Tables

**Table 1:** Sequence information of 10 Kunitz peptides.

Peptide Name	Unigene_id	Sequences
DAKS1	Unigene7405_D_acutus	AVCSQEAMTGPCRAVMPRWYFDMYKKKCIRFIYGGCGGNRNNFESEEYCMAVCKKMI
DAKS2	Unigene2097_D_acutus_1	DRCHSSKMVGPCRASFHRWWYNATSQTCQEFIFGGCKGNANNFFSEQDCFQTCARVA
DAKS3	Unigene2097_D_acutus_2	EFCAAPQMVGPCRASFHRWYFDLESRTCKMFVYGGCHGNKNNYLFEEHCWNQCTGDG
DAKS4	Unigene24735_D_acutus_2	DLCQLPQEEGPCDLKLPRWFYNPKTDRCQKFDFGGCAGNANNFKRRRWCRLRCRKR
DAKS5	Unigene5437_D_acutus	DICQLKKEDGPCRNFVLKWYFDPKTASCARFWYGDCGGNENRFNTQKDCEKVCVSAH
DAKS6	Unigene2946_D_acutus_2	SYCVDMPDTGPCEGSLPRWYYNPLTEKCGRFTYGGCEGNKNNFEQEETCMEICRGVT
DAKS7	Unigene2946_D_acutus_1	DHCLAPYKVGRCRGSFHRWYYNPEIEQCQQFIFGGCNPNKNNYVRKEECNLACKKVG
DAKS8	Unigene20491_D_acutus_2	AFCLLENDPGVCRGLIPRYFYNKESQQCEKFQYGGCLGNQNNFKSLQECQNTCHDRV
DAKS9	Unigene20491_D_acutus_1	SICALKADSGPCKALHTRYHFDIHTRHCESFNFGGCQGNENNFLTLEECQQTCVIKD
DAKS10	Unigene20491_D_acutus_3	SLCVMPMDRGLCKANEKRFFYNQTTGRCRPFSYTGCGGNENNFTSRKACVQM

**Table 2:** Inhibitory activity of DAKS1-10 against FXIa.

Peptide Name	Concentration (uM)	Inhibition Rate (%)
DAKS1	7.5	98.13 ± 0.31
	0.15	91.20 ± 0.31
	0.05	63.51 ± 0.96
DAKS2	7.5	7.54 ± 0.34
DAKS3	7.5	14.56 ± 0.45
DAKS4	7.5	65.26 ± 1.10
	0.15	9.82 ± 0.32
	0.05	4.54 ± 1.18
DAKS5	7.5	−14.24 ± 1.08
DAKS6	7.5	−8.840 ± 0.75
DAKS7	7.5	6.237 ± 1.92
DAKS8	7.5	−8.820 ± 2.12
DAKS9	7.5	69.07 ± 2.08
	0.15	27.16 ± 0.98
	0.05	16.17 ± 1.28
DAKS10	7.5	−10.03 ± 1.24

Inhibition of DAKS1-10 against FXIa assessed in chromogenic substrate assay. Results obtained from 3 independent experiments.

**Table 3:** Protease specificity of DAKS1 and PN2KPI.

Inhibitor	Molar Ratio	Inhibition, %
DAKS1	1:125, FXIa:inhibitor	>70
PN2KPI	1:13.3, FXIa:inhibitor	>90
DAKS1	1:6000, FXa:inhibitor	27.45
PN2KPI	1:504, FXa:inhibitor	55.84
DAKS1	1:6000, thrombin:inhibtor	1.54
PN2KPI	1:2066, thrombin:inhibtor	−39.51
DAKS1	1:3000, kallikrein:inhibitor	62.28
PN2KPI	1:206, kallikrein:inhibitor	16.65
DAKS1	1:500, trypsin:inhibitor	68.46
PN2KPI	1:52, trypsin:inhibitor	87.41
DAKS1	1:6000, FXIIa:inhibitor	0
PN2KPI	1:1033, FXIIa:inhibitor	19.58

## Data Availability

Data is contained within the article and supplementary material.

## Supplementary Materials

The following are available online at https://www.mdpi.com/article/10.3390/ph14100966/s1. Figure S1: alignment of amino acid sequence of Kunitz domains from venom of *D. acutus*; Figure S2: DAKS1 inhibited trypsin; Figure S3: PN2KPI inhibited FeCl3-induced arterial thrombosis formation; Table S1: information of 11 proteins containing Kunitz domains from the annotated transcriptome library of the venom of *D. acutus.*
