# Supplementary material for: DAKS1, a Kunitz Scaffold Peptide from the Venom Gland of Deinagkistrodon acutus Prevents Carotid-Artery and Middle-Cerebral-Artery Thrombosis via Targeting Factor XIa
Source: Pharmaceuticals (Basel). 2021 Sep 24;14(10):966. doi: 10.3390/ph14100966 (PMC8539665; doi:10.3390/ph14100966)
Supplement: Supplementary file 1 [file pharmaceuticals-14-00966-s001.zip › pharmaceuticals-1366247 SUPPL/pharmaceuticals-1366247 suppl.pdf]

# Supplementary Information

## Supplementary Figures

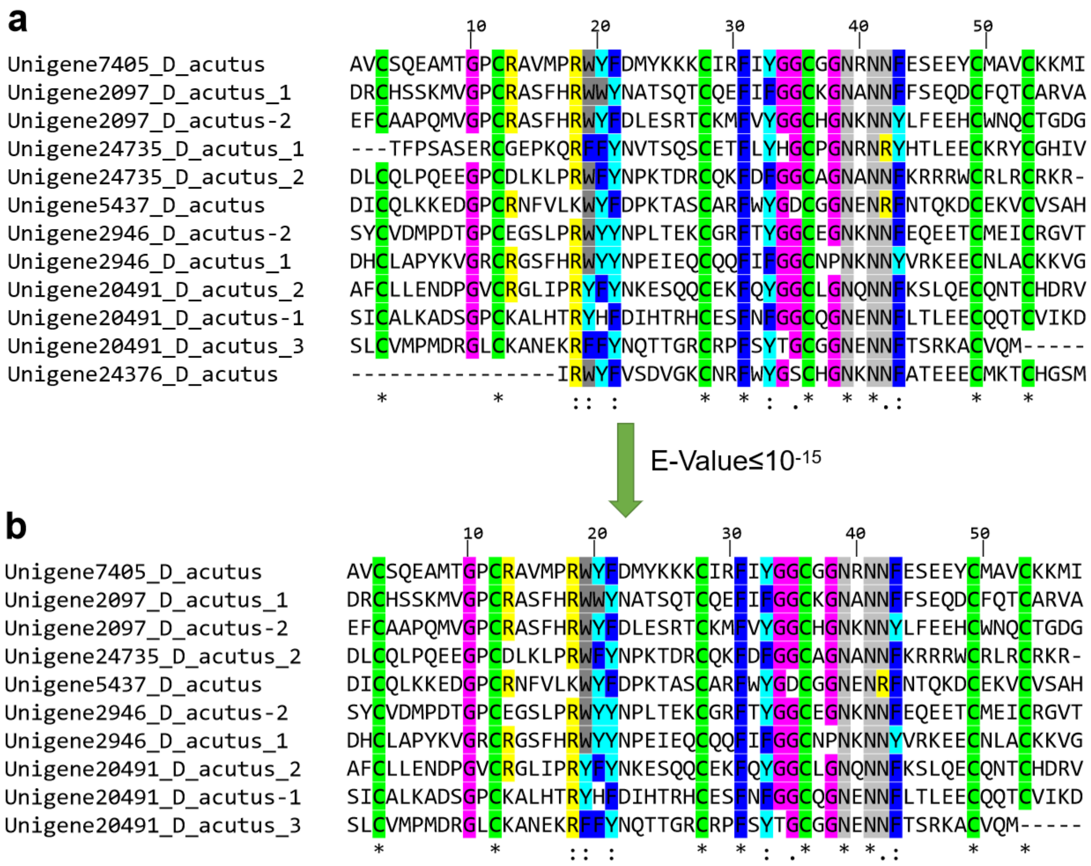


**Figure S1.** Alignment of amino acid sequence of Kunitz domains from venom of *D. acutus*. **(a)** The multiple sequence alignments of 12 Kunitz scaffold-based peptides recognized by the Pfam database. **(b)** The multiple sequence alignments of 10 Kunitz scaffold-based peptides recognized by the Pfam database when the E-Value is below $10^{-15}$. "*" indicates positions with fully conserved residue. ":" indicates conservation between sequences with similar properties and "." indicates conservation between groups of weak similar properties.

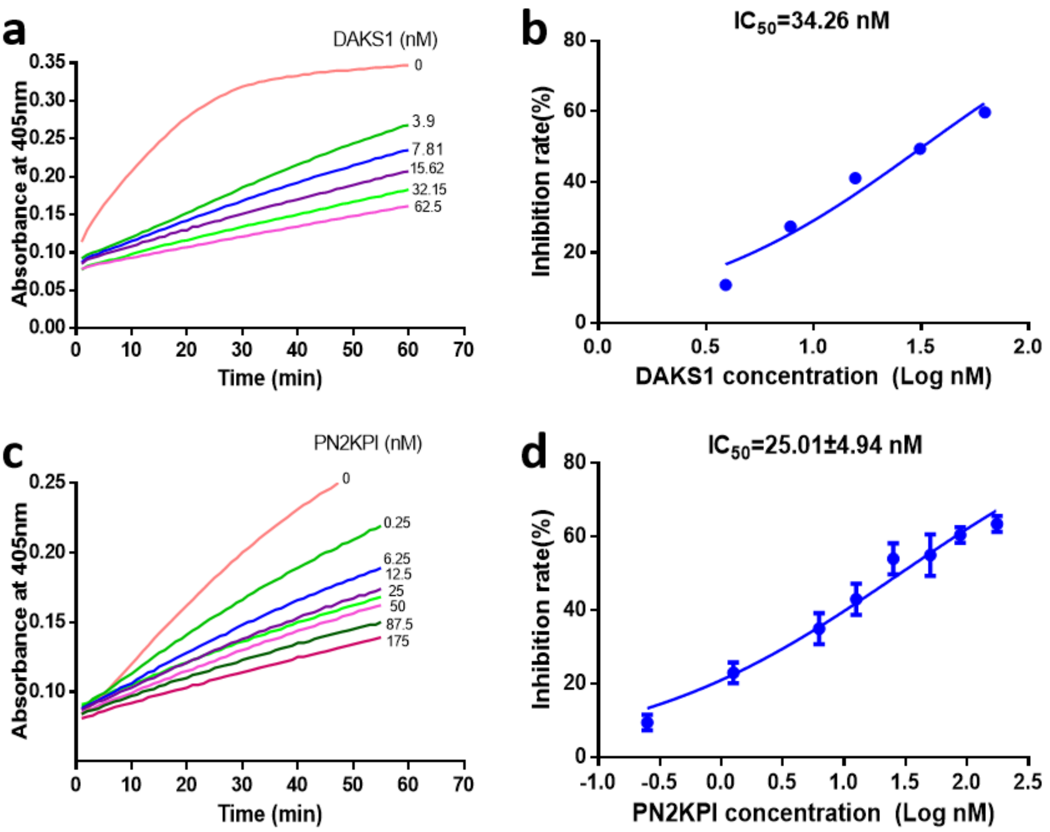


**Figure S2.** DAKS1 inhibited Trypsin. **(a)** The Inhibition activity of DAKS1 towards Trypsin. Reactions started by addition of CS-11(22) (250 μM) to a mixture containing DAKS1 (0-125 nM) incubated for 60 mins with Trypsin (4.15 nM). Substrate was hydrolyzed monitored at 405 nm for 60 mins. (**b)** Determination of the $IC_{50}$ of DAKS1. Plot of inhibition rate (%) vs log DAKS1 concentrations was fitted by nonlinear regression and the value of $IC_{50}$ was obtained by fit log(inhibitor) vs. normalized response-variable slope. (**c)** The Inhibition activity of PN2KPI towards Trypsin. Reactions started by addition of CS-11(22) (250 μM) to a mixture containing PN2KPI (0-175 nM) incubated for 60 mins with Trypsin (4.15 nM). Substrate was hydrolyzed monitored at 405 nm for 60 mins. (**d)** Determination of the $IC_{50}$ of PN2KPI. Plot of inhibition rate (%) vs log PN2KPI concentrations was fitted by nonlinear regression and the value of $IC_{50}$ was obtained by fit log(inhibitor) vs. normalized response-variable slope. Data are presented as the mean ± SD of three independent experiments.

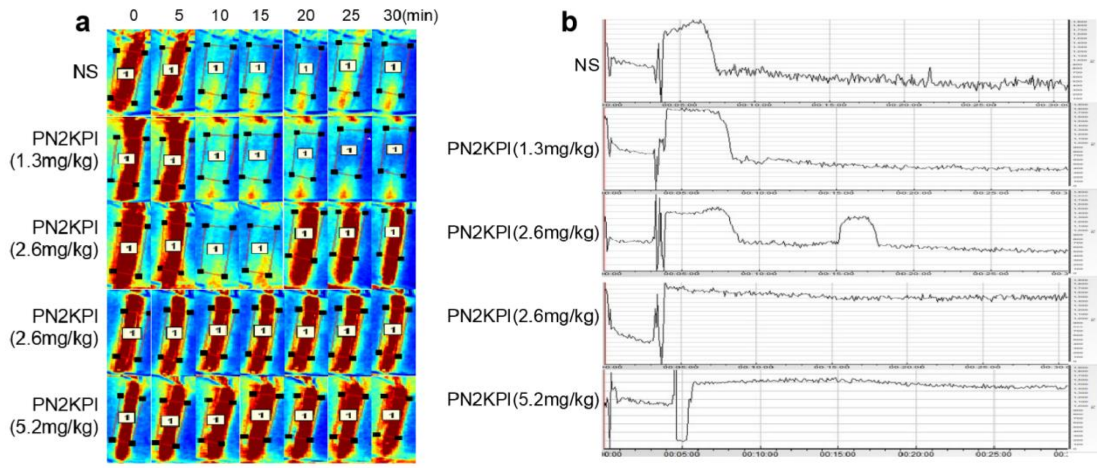


**Figure S3.** PN2KPI inhibited $FeCl_3$-induced arterial thrombosis formation. (**a**) Saline and PN2KPI(1.3, 2.6 and 5.2mg/kg) were injected through a tail vein 10 min before $FeCl_3$-induce, and blood flow at region of interest (ROI) was monitored at 0, 5, 10, 15, 20, 25 and 30 min. (**b**) Representative blood perfusion graphs of mice treated with ,NS, PN2KPI. NS: Normal Saline.
